# A school-family blended multi-component physical activity program for Fundamental Motor Skills Promotion Program for Obese Children (FMSPPOC): protocol for a cluster randomized controlled trial

**DOI:** 10.1186/s12889-023-15210-z

**Published:** 2023-02-20

**Authors:** Lin Zhou, Wei Liang, Yuxiu He, Yanping Duan, Ryan E. Rhodes, Sonia Lippke, Julien S. Baker, Yu Liang, Lin Han, Wan Xin Liu, Qi Liu

**Affiliations:** 1 School of Physical Education, Hebei Normal University, Shijiazhuang, China; 2 Key Laboratory of Measurement and Evaluation in Exercise Bioinformation of Hebei Province, Hebei, Wuhan, China; 3 College of Physical Education, Shenzhen University, Shenzhen, China; 4 Department of Sport, Physical Education and Health; Hong Kong Baptist University, Kowloon, Hong Kong; 5 School of Exercise Science, Physical and Health Education, University of Victoria, Victoria, Canada; 6 Constructor University Bremen (formerly known as Jacobs University Bremen), Bremen, Germany; 7 Shenzhen Sports School, Shenzhen, China; 8 The Education University of Hong Kong, Ting Kok, Hong Kong

**Keywords:** Health, Fundamental motor skill, Children, Obesity, Physical activity, Blended intervention, Behavioral change techniques (BCT), RE-AIM, Implementation science

## Abstract

**Background:**

Fundamental motor skills (FMSs) are crucial for children’s health and comprehensive development. Obese children often encounter a considerable challenge in the development of FMSs. School-family blended PA programs are considered a potentially effective approach to improve FMSs and health-related outcomes among obese children, however, empirical evidence is still limited. Therefore, this paper aims to describe the development, implementation, and evaluation of a 24-week school-family blended multi-component PA intervention program for promoting FMSs and health among Chinese obese children, namely the Fundamental Motor Skills Promotion Program for Obese Children (FMSPPOC) employing behavioral change techniques (BCTs) and building on the Multi-Process Action Control (M-PAC) framework as well as using the Reach, Effectiveness, Adoption, Implementation, and Maintenance (RE-AIM) framework for improving and evaluating the program.

**Methods:**

Using a cluster randomized controlled trial (CRCT), 168 Chinese obese children (8–12 years) from 24 classes of six primary schools will be recruited and randomly assigned to one of two groups by a cluster randomization, including a 24-week FMSPPOC intervention group and a non-treatment waiting-list control group. The FMSPPOC program includes a 12-week initiation phase and a 12-week maintenance phase. School-based PA training sessions (2 sessions/week, 90 min each session) and family-based PA assignments (at least three times per week, 30 min each time) will be implemented in the initiation phase (semester time), while three 60-min offline workshops and three 60-min online webinars will be conducted in the maintenance phase (summer holiday). The implementation evaluation will be undertaken according to the RE-AIM framework. For intervention effectiveness evaluation, primary outcome (FMSs: gross motor skills, manual dexterity and balance) and secondary outcomes (health behaviors, physical fitness, perceived motor competence, perceived well-being, M-PAC components, anthropometric and body composition measures) will be collected at four time-points: at baseline, 12-week mid-intervention, 24-week post-intervention, and 6-month follow-up occasions.

**Discussion:**

The FMSPPOC program will provide new insights into the design, implementation, and evaluation of FMSs promotion among obese children. The research findings will also supplement empirical evidence, understanding of potential mechanisms, and practical experience for future research, health services, and policymaking.

**Trial registration:**

Chinese Clinical Trial Registry; ChiCTR2200066143; 25 Nov 2022.

**Supplementary Information:**

The online version contains supplementary material available at 10.1186/s12889-023-15210-z.

## Contributions to the Literature

- This study will guide future development, implementation and evaluation of FMSs promotion programs among obese children in the long run
- This study will provide a paradigm of integrating theories and implementation strategies, adding values to future research and practice in health promotion among obese children
- Research findings will supplement empirical evidence and practical experience for future implementation science on addressing FMSs, healthy lifestyles, and physical and mental health among obese children
- Research findings will add more understandings of the underlying mechanism of FMSs development, contributing to future theory refinement, intervention design and policymaking

## Introduction

Fundamental motor skills (FMSs) are considered the building blocks for more advanced and complicated movements (e.g., games, sports, and recreational activities) that children will develop throughout their lives [1]. FMSs represent a degree of proficiency in a range of motor skills as well as underlying mechanisms such as motor coordination and control [1–3]. Commonly developed in childhood and subsequently refined into context- and sport-specific skills, FMSs can be categorized as three aspects: *locomotor skills* (e.g., running, jumping, and hopping), *object control/ball/manipulative skills* (e.g., throwing, catching, and dribbling), and *stability skills* (non-locomotor, e.g., balancing and twisting) [4, 5]. The mastery of FMSs has been purported as crucial elements of children’s physical, social and psychological development, which occurs in an orderly and sequential manner [6, 7]. Good FMSs may be the foundation towards a healthier life because of positive connections with physical activity (PA) [8–10], contributing to greater physical fitness [2, 4, 11], body weight status [12, 13], perceived motor competence [10, 14], sports engagement [15], cognitive function [16, 17], perceived well-being [18], and perceived quality of life [19].

Despite the important role of FMSs in children’s holistic development, obese children are usually confronted with an accelerated challenge in developing FMSs [20]. There have been numerous studies indicating that obese children were delayed in FMSs development and showed a prominently poorer performance of FMS tests compared with their peers with healthy weight [21–25]. This may be attributed to multifaceted factors. For example, large body mass index (BMI) can lead to excessive pressure to children’s skeletal system [26] and unfavorable changes in the major brain sites of neuroplasticity [27, 28], and decrease perceived motor capabilities [29], which subsequently inhibit the development of children’s FMSs. For obese children, poor FMSs may also weaken their motivation for engaging in PA and result in a low level of physical fitness, which in turn deteriorates adiposity status and causes a series of negative consequences for other health aspects, e.g., metabolic diseases, and mental disorders among children [30–32]. This vicious circle has been clearly illustrated in Stodden et al.’s model [33], which to some extent emphasizes the importance and necessity of promoting FMSs in obese children.

Traditionally, PA intervention programs have shown effectiveness in improving FMSs [32] and other health-related outcomes (e.g., physical fitness, cognition, and mental health) in overweight and obese children [34–36]. Schools are considered an ideal setting to implement PA interventions with the aim of promoting FMSs, as children spend most of their waking hours at school and schools can also provide better conditions (e.g., facilities, equipment, curriculum, health experts, peer support), easier access and maximum reach for intervention efforts [37, 38]. However, school-alone settings cannot address the large amount of out-of-school time (e.g., summer holidays) and parental influences that shape children’s behaviors in the home setting [39]. By contrast, family-based PA programs usually focus on the impacts of parents’ support, knowledge, attitudes, motivation, and other psychosocial factors towards children’s behaviors and emphasize the co-activity of parents and children to promote children’s FMSs development [37, 40, 41]. Previous studies have also provided evidence for the effectiveness of family-based PA interventions on improving children’s FMSs and healthy behavioral patterns [40, 41]. Nevertheless, for obese children, the development of FMSs requires more professional instruction and practice guided by qualified experts [3, 42], and this cannot be fully guaranteed in family-alone interventions. In addition, a social cooperative and interactive environment (e.g., at school) can facilitate a better development of FMSs, PA self-efficacy and social skills for obese children [43], while this cannot be fully achieved in a family-alone setting. Taken together, this suggests that a school-family blended PA intervention paradigm which can combine the merits of both school-alone and family-alone approaches, shows great potential in promoting FMSs and related health outcomes among obese children.

Notwithstanding the advocation of school-family integrated PA interventions, empirical evidence on obese children is still scarce, especially in China [44]. In addition, several limitations and research gaps of previous FMSs promotion programs should be further addressed. For example, previous studies show inconsistent findings in the intervention effect on balance among obese children [45, 46], which may be attributed to the lack of specific PA sessions that are tailored to promoting children’s balance in some studies [32]. Furthermore, the findings in terms of the long-term effects of PA interventions on obese children’s FMSs were mixed in previous studies [32]. Some studies indicated a sustained intervention effect of PA intervention on FMSs (e.g., at the 36-week follow-up assessment) [47], while others found that the increase in FMSs of obese children was not maintained during the follow-up, which even relapsed back to baseline levels [48–50]. The underlying reasons may be the absence of theory-based targets of interventions and Behavioral Change Techniques (BCT) that play a crucial role in maintaining the PA intervention effects in the long run [51, 52]. Moreover, the mechanisms of why the PA intervention successfully improved obese children’s FMSs have not been systematically examined in previous studies [53]. Identifying the mediation and moderation mechanisms of the intervention program is important and necessary, as it contributes to future design of effective FMSs promotion programs. In addition, previous studies have also shown a series of methodological limitations (e.g., lack of randomization and blinding, lack of objective standardized measures, lack of comprehensive evaluations for the intervention fidelity and quality) [32, 53], which may weaken the future implementation and generalization of the study findings.

To address the research and practice gaps, the present study aims to develop, implement, and evaluate a 24-week school-family blended multi-component PA intervention program for promoting FMSs and health among Chinese obese children, namely Fundamental Motor Skills Promotion Program for Obese Children (FMSPPOC). The particular objectives of outcome evaluation include: (1) examine the intervention effects of the FMSPPOC on improving the primary outcome (i.e., FMSs) among Chinese obese children; (2) examine the intervention effects of the FMSPPOC on improving the secondary outcomes (health behaviors, physical fitness, perceived motor competence, perceived well-being, M-PAC components, anthropometric and body composition measures) among Chinese obese children; and (3) identify the interrelationships between FMSs and physical and psychological outcomes (i.e., mediation mechanisms) and the moderating role of demographics.

## Method

### Study design

**Fig. 1:**
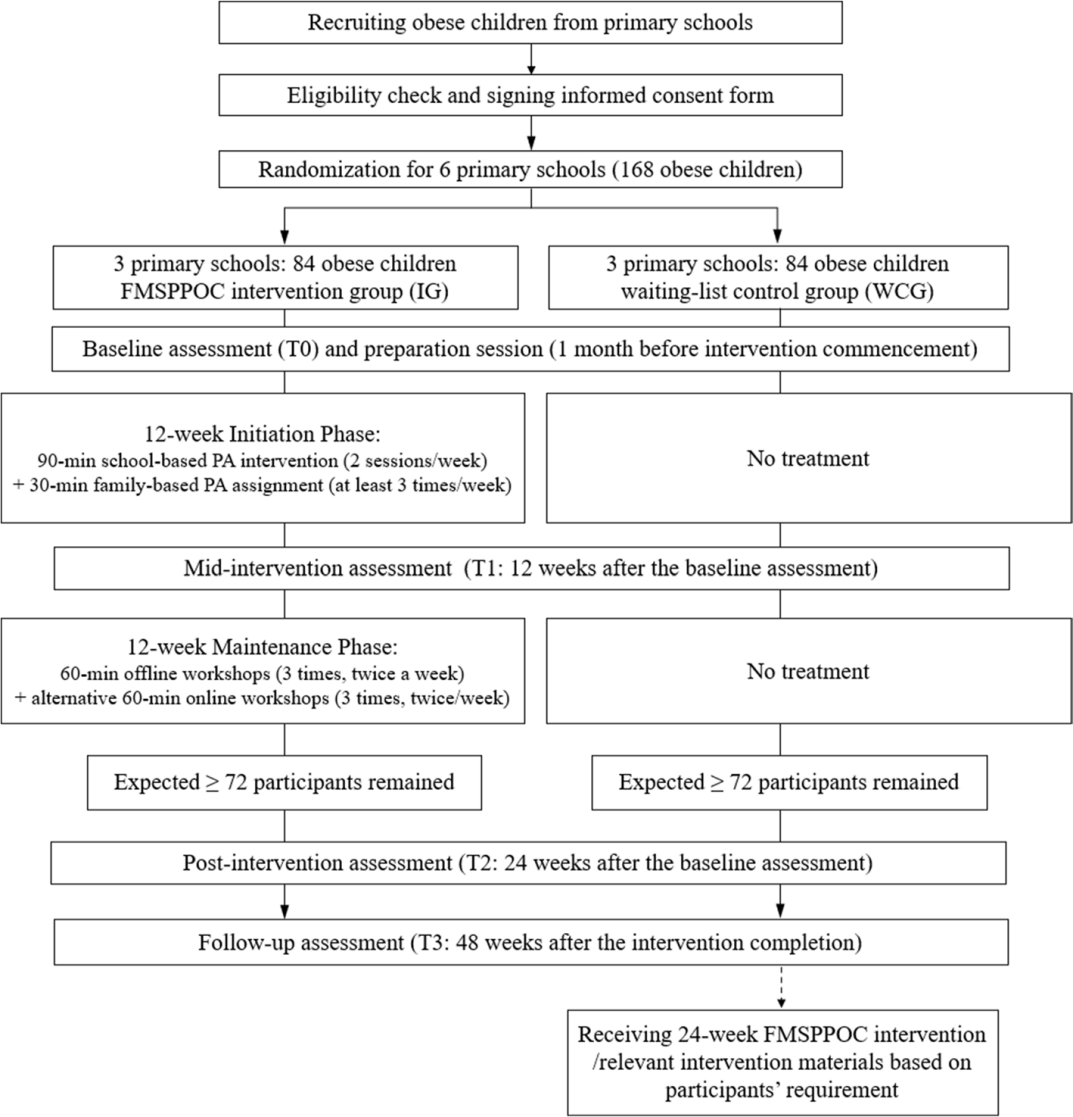
CONSORT flow diagram

This study will apply a two-group double-blinded cluster randomized controlled trial (CRCT) with four measurement occasions, including baseline assessment (T0), mid-intervention assessment (T1: 12 weeks after the baseline assessment), post-intervention assessment (T2: 24 weeks after the baseline assessment), and follow-up assessment (T3: 48 weeks after the baseline assessment). Two groups include: 1) an intervention group (IG), receiving the FMSPPOC intervention for 24 weeks; and 2) a waiting-list control group (WCG), receiving the FMSPPOC intervention or relevant materials (based on participants’ requirements) after the completion of all data collection for IG (see Fig. 1). The design, implementation, and reporting of the FMSPPOC will follow the SPIRIT guidelines and the CONSORT statement [54, 55] (Supplement Material 1). The study protocol was approved by the Research Ethics Committee of Hebei Normal University (ref. No.2021LLSC051). This study was a key part of a large research project funded by the National Social Sciences Funding Committee of China (Ref. No.: 19200526).

### Participants

As childhood is a critical period for developing favorable FMSs that can continuously affect health and individual development in adolescence and adulthood, a health-promoting intervention that targets this age group is vitally important [56]. In pre-adolescence, neuroplasticity may be greater, changes faster based on experience [57, 58] and the brain may be particularly sensitive to the effects of PA when the brain's neural circuits are still developing [59]. Therefore, to address age-related declines in PA and maximize the benefits of PA on FMSs, obese children aged 8 to 12 years were selected as the target population for this study with obesity defined as a body mass index (BMI) at or above the 95^th^ percentile of the sex-specific BMI-for-age growth charts (https://www.cdc.gov/obesity/data/childhood.html).

#### Sample size estimate

An average medium effect size (Cohen *d* = 0.54) of PA intervention on FMSs in obese children was proposed based on previous studies [32]. Considering the minimal class size is 30 and the prevalence rate of childhood obesity is approximately 20% [60], a cluster size of 6 (for each class) was selected in this study. Using an ICC of 0.01 [61], an alpha of 0.05, a statistic power of 80%, and an attrition rate of 15% [43], at least 84 participants in each condition (12 clusters per condition), for a total of 168 participants in 24 clusters will be required for this CRCT.

#### Recruitment and eligibility criteria

Based on the sample size estimate, this study is intended to recruit 24 classes from six primary schools (grades 2–5), excluding the private or special education schools or those participating in other PA-related programs. Using a random stratified sampling approach (e.g., socioeconomic status, geographical location, grade and class size of each school), six primary schools will be recruited in Shijiazhuang, Hebei, China. An invitation letter describing the study nature and participation requirement will be delivered to the principals of eligible schools. Upon the approval of the principals, one class from each grade will be randomly selected to participate in this study. All children in the selected classes will be provided with an informed pack comprising a plain language statement and a written informed consent form to be signed by their parents. The eligibility criteria will include: (1) aged 8–12 years; (2) Body Mass Index (BMI) greater than Chinese obesity cutoffs corresponding to 95th percentile of sex-specific and age-specific BMI reference standards [62]; (3) no previous substantial experiences in participating in FMSs-related promotion programs; (4) no prior diagnosis of physical, verbal, and cognitive disorders that may prevent participation in PA program and interrupt the outcome evaluation.

#### Randomization and blinding

To avoid the potential for contamination, randomization will be conducted at the school level. All eligible schools will be randomly assigned to one of the two groups prior to baseline assessment, where each group will consist of three schools. Each school will select 28 obese children from four classes covering grades 2–5 to participate in this study, and the children in the same school will receive the same treatment. Randomization will be implemented with a ratio of 1:1, using the Excel software by a researcher who will not be involved in the participant recruitment, data collection or evaluation. Due the ethical concerns, participants are not able to be blinded as they will be informed with the study purpose and group allocation in the written informed consent form. Intervention facilitators and outcome evaluators will be concealed for the group allocation.

### Fundamental motor skills Promotion Program for Obese Children (FMSPPOC)

FMSPPOC is a school-family blended multi-component intervention program, which aims to promote the development of obese children’s FMSs in a supportive environment, providing the children with both positive experiences (e.g., enjoyment, success, and accomplishment) in relation to PA, and cultivate interest in sports, so as to enhance their PA level/engagement, perceived motor competence, fitness and wellbeing in future. The FMSPPOC will last 24 weeks, consisting of two parts: a 12-week initiation phase, and a 12-week maintenance phase.

#### Theoretical backdrop: multi-process action control model

To enhance the effectiveness and implementation of the intervention, the Multi-Process Action Control model (M-PAC) will be used as the theoretical backdrop [63]. The M-PAC model postulates that individuals’ behavioral change is a continued process from intention formation to behavioral initiation and maintenance, consisting of reflective, regulatory, and reflexive processes [64]. In the M-PAC framework, intention is conceived as a decisional construct (i.e., has intention/ has no intention). Similar with the tenets of other psychosocial theories (e.g., the Theory of Planned Behavior, TBP; Capability, Opportunity, Motivation, Behavior model, COM-B), the M-PAC emphasizes the influence of several determinants of intention which function in the reflective processes (i.e., consciously deliberated and expected consequences of performing a behavior) [65]. Particularly, instrumental attitude (e.g., PA is useful), affective attitude (e.g., PA is enjoyable), perceived capability (e.g., I have the ability to perform a behavior), and perceived opportunity (e.g., I have the time and can access to perform PA) play a crucial role in forming a behavioral intention [66]. Furthermore, the M-PAC framework proposes that whether a behavioral intention can be successfully translated to an actual behavior is determined by the reflective processes of affective attitude and perceived opportunity as well as the enactment of regulation processes. Particularly, higher levels of affective attitude and perceived opportunity are considered necessary for successful translation of behavioral intention into actual behavior than for intention formation. Similar with the Health Action Process Approach (HAPA), the M-PAC framework also suggests the importance of regulatory strategies (e.g., action planning and coping planning) in the intention-behavior translation [67]. In addition, extending on previous psychosocial theories, the M-PAC framework highlights the important role of diverse impulsive components in behavioral maintenance (i.e., “continuance of actional control is thought to rely upon the development of reflexive processes”) [68]. It suggests that impulsive components affect actional control most often through learned associations and are triggered through specific circumstances/cues and stimuli [69]. The M-PAC framework emphasizes the development of two crucial reflexive processes, including habit (e.g., I will engage in PA automatically) and identity (e.g., I am a person who is physically active), as individuals begin to perform the behavior more regularly [63–65]. Therefore, a developed behavioral pattern of action control will be determined by the independent influence of relative, regulatory, and reflexive processes [63–65]. Targeting the psychosocial components of the M-PAC model, a series of Behavioral Change Techniques (BCTs) will be also adopted [70, 71] in the FMSPPOC program (see Table 1).

**Table 1:** M-PAC components and behavioral change techniques involved in the FMSPPOC

Approach	Time	Target objects	M-PAC components	Behavioral change techniques (BCT) addressed	Examples
Briefing sessions & Education materials	Week 1, 2, 12 & 13	Parents and children	Perceived capability; Instrumental attitude; Perceived opportunity; Affective attitude	- Instruction on how to perform a behavior (4.1) - Information about health consequences (5.1) - Information about social and environmental consequences (5.3 & 5.6) - Information on others’ approval (6.3) - Restructuring the physical environment (12.1) - Prompts/cues (7.1) - Feedback on behavior (2.2)	Introducing the benefits of participating in PA regularly, and the negative consequences of physical inactive; Introducing the importance of FMSs, especially for obese children; Introducing the feasible and pragmatic approaches (e.g., PA) to develop FMSs; Introducing the nature and content of the FMSPPOC program
School-based PA sessions delivered by PE teachers	Week 1–12	Children	Perceived capability; Instrumental attitude; Perceived opportunity; Affective attitude	- Instruction on how to perform a behavior (4.1) - Behavioral practice (8.1) - Feedback on behavior (2.2) - Verbal persuasion (15.1) - Social comparison (6.2) - Prompts/cues (7.1)	PE teachers will teach participants how to play the ball games, guide them to practice, and provide them with immediate feedback; Participants will learn and practice the PA sessions; Group activities will be implemented during the PA sessions; PE teachers and peers will provide verbal encouragement; A weekly peer role model will be selected by participants and teachers
Family-based PA assignments completed by both parents and children	Week 1–12	Parents, children, and other social networks	Perceived opportunity; Affective attitude; Behavioral regulation	- Restructuring the physical environment (12.1) - Prompts/cues (7.1) - Behavioral practice (8.1) - Feedback on behavior (2.2) - Practical support (3.2) - Emotional support (3.3) - Problem solving (1.2) - Action planning (1.4) - Goal setting (behavior) (1.1) - Self-monitoring of behavior (2.3)	Parents will be taught how to establish a favorable PA environment at home; Parent–child co-activity will be implemented; Parents will be asked to provide their child/children with verbal encouragement; A short-term goal and a long-term goal will be set; Incentive will be provided based on the engagement of family-based PA assignment; Ranking games will be implemented; Parents will be asked to monitor the child/children’s PA performance and upload on the website
Online and offline workshops	Week 14, 16, 18, 20, 22 & 24	Parents and children	Perceived capability; Instrumental attitude; Perceived opportunity; Affective attitude; Behavioral regulation; Identity; Habit	- Information about health consequences (5.1) - Information about social and environmental consequences (5.3 & 5.6) - Information on others’ approval - Behavioral practice (8.1) - Feedback on behavior (2.2) - Problem solving (1.2) - Review about goals (1.5) - Self-monitoring of behavior (2.3) - Behavioral substitution (8.2) - Habit formation (8.3) - Unspecified support (3.1) - Practical support (3.2) - Emotional support (3.3) - Prompts/cues (7.1)	Relevant knowledge such as long-term development of FMSs, benefits and consequences of healthy lifestyles, and potential risks of FMSs development will be delivered; Acute PA practice will be implemented; Problems will be discussed during the workshops and solutions will be confirmed by experts, PE teachers, parents and child/children on consensus; Feedback on behaviors and goals will be provided by PE teachers, experts and parents; Values and meaning of the activity as well as other tactics (e.g., visual and tactile symbols) of the new identity will be emphasized

#### 12-week initiation phase

During the first 12-week initiation phase, the intervention consists of school-based PA sessions and family-based PA assignments for the whole classes including the obese children to prevent stigmatization or exclusion. Particularly, the school-based PA sessions will be delivered twice a week (90 min each session) for 12 weeks (totally 24 sessions). Three ball games (i.e., soccer, basketball, and volleyball) will be used as the main content of school-based PA sessions for FMSs promotion due to the consideration of effectiveness, enjoyment, feasibility, and greater adherence [72, 73]. Each session will include a 5-min warm-up, a 15-min physical fitness training, a 60-min FMSs training (30-min learning and practice + 30-min game), and a 10-min cool-down. For the FMSs training part, three types of ball games will be delivered in different weeks, including week 1–4 for soccer, week 5–8 for basketball, and week 9–12 for volleyball. Each ball game section will include skill instruction and learning, skill practice (guided by teachers + self), game part, and review/summary. All the movements in each section will be designed to achieve a moderate-to-vigorous intensity. Detailed content of the school-based PA sessions can be found in Table 2.

**Table 2:** The content of the school-based PA sessions

PA-sessions (Time allocation)	Warm-up (5-min)	Physical fitness training (15-min)	FMSs training (60-min)		Cool-down (10-min)
			30-min learning & practice	30-min game	
**Week 1–4:** Soccer FMSs training	50–60% HR_max_ Jogging, dynamic stretching of limbs and trunk muscles The PE teacher will briefly introduce the course content and objectives during the warm-up	70–90%HR_max_ 2–3 sets of combined exercise: 1) 5 × 10 shuttle run; 2) 50 m sprint 3) 10–15 push-ups 4) 20–30 deep squats; 5) 20–30 sit-ups; 6) 30-60 s basic hip bridge; 7) 30-60 s plank; 8) 30-60 s jumping jacks; 9) 30-60 s high knees The PE teacher will select 6–8 movements to practice; During the interval, 1-min mark time will be performed during the recovery interval	70–90%HR_max_ Practice in small groups: 1) Off-ball; 2) Throw-Ins; 3) Simple dribbling, passing and shooting; 4) Trapping; 5) Juggling	70–90%HR_max_ Group-based game: 1) Dribble game; 2) Juggling soccer game; 3) Spot shooting game; 4) Small-size soccer match: 3 vs 3 or 5 vs 5	50–60% HR_max_ Dynamic stretching and relaxation of the limbs and trunk muscles Teachers will introduce common knowledge or stories related to soccer, basketball, and volleyball according to the progress of the PA sessions Participants will be asked to share their feelings about the PA sessions
**Week 5–8:** Basketball FMSs training			70–90%HR_max_ Practice in small groups: 1) Passing (e.g., chest, push and bounce) and catching; 2) Dribbling (single or both hands); 3) Shooting (spot or move); 4) Foot work; 5) Layups (both hands)	70–90%HR_max_ Group-based game: 1) Fixed-point shots; 2) Passing and catching games; 3) Dribbling relays; 4) Small-size basketball match: 3 vs 3 or 5 vs 5	
**Week 9–12:** Volleyball FMS training			70–90%HR_max_ Practice in small groups: 1) Multi-directional forms of running, jumping, and skipping; 2) Passing (e.g., proper hand placement, proper stance, dig pass); 3) Serving (e.g., practice serving the ball against the wall or partner); 4) Setting (e.g., pass the ball to a teammate or over the net to the opposing team); 5) Triangle passing drill	70–90%HR_max_ Group-based game: 1) Dig pass game; 2) Dig pass over the net game; 3) Serve and run drill game 4) Butterfly passing drill game	

In addition to school-based PA training, all the participants are asked to complete a 30-min family-based PA assignment at least three times per week during the first 12-week initiation phase. The purpose of the family-based PA assignment is to reinforce FMSs practice and promote PA engagement during the out-of-school time. For each PA assignment, participants are asked to complete several activities together with their parents (i.e., parent–child co-activity). These activities are modified from the school-based PA sessions that can be easily undertaken in a family-setting (e.g., require minimal equipment, can be undertaken indoors or outdoors within limited space). Parents will also be asked to track their child/children’s weekly completion times and conditions and upload this information to a designated column of the Ding-Talk APP (https://www.dingtalk.com/en, Alibaba Group). At weekends, the research team members will check the participants’ PA assignments and reward them with small red flower stickers as incentives: participants who complete PA assignments three times will receive one sticker while who complete the assignments five times or more will receive two stickers. The greater number of red flowers participants accumulates, the more prizes (e.g., sports bracelets, suits, and sneakers) they can redeem at the end of the intervention.

#### 12-week maintenance phase

12-week maintenance phase will be implemented mainly during the summer holidays following the 12-week initial phase. This part will consist of three face-to-face workshops with three online webinars (using Ding-Talk APP) only for the target population of obese children. The offline workshops and online webinars will be conducted alternatively and biweekly. Each workshop and webinar will last for 60 min, consisting of 30-min expert talk, 20-min interactive activity, and 10-min Q&A. The main purposes of the offline workshops and online webinars will include: (1) maintaining children’s FMSs practice; (2) promoting healthy lifestyles (e.g., regular engagement of PA, limited sedentary time, high quality sleep, balanced diet) in the long run; (3) addressing existing problems and future challenges. The topics will include (1) practicing FMSs in daily life (e.g., FMSs games introduction, tips of FMSs practice); (2) PA and health (e.g., recommendation of PA for children, co-activities of parent and child); (3) sedentary behavior and health (e.g., recommendation of screen time, tips of interrupting prolonged sitting); (4) good sleep (e.g., sleep recommendation in terms of the duration and quality); (5) eating happily and healthily (e.g., healthy food selection, healthy cooking, and “say no to snacks”); (6) existing problems and future challenges in children’s FMSs (e.g., existing problems in daily practice of FMSs, how to maintain the development of FMSs after the project completion).

### Procedure and quality control

**Fig. 2:**
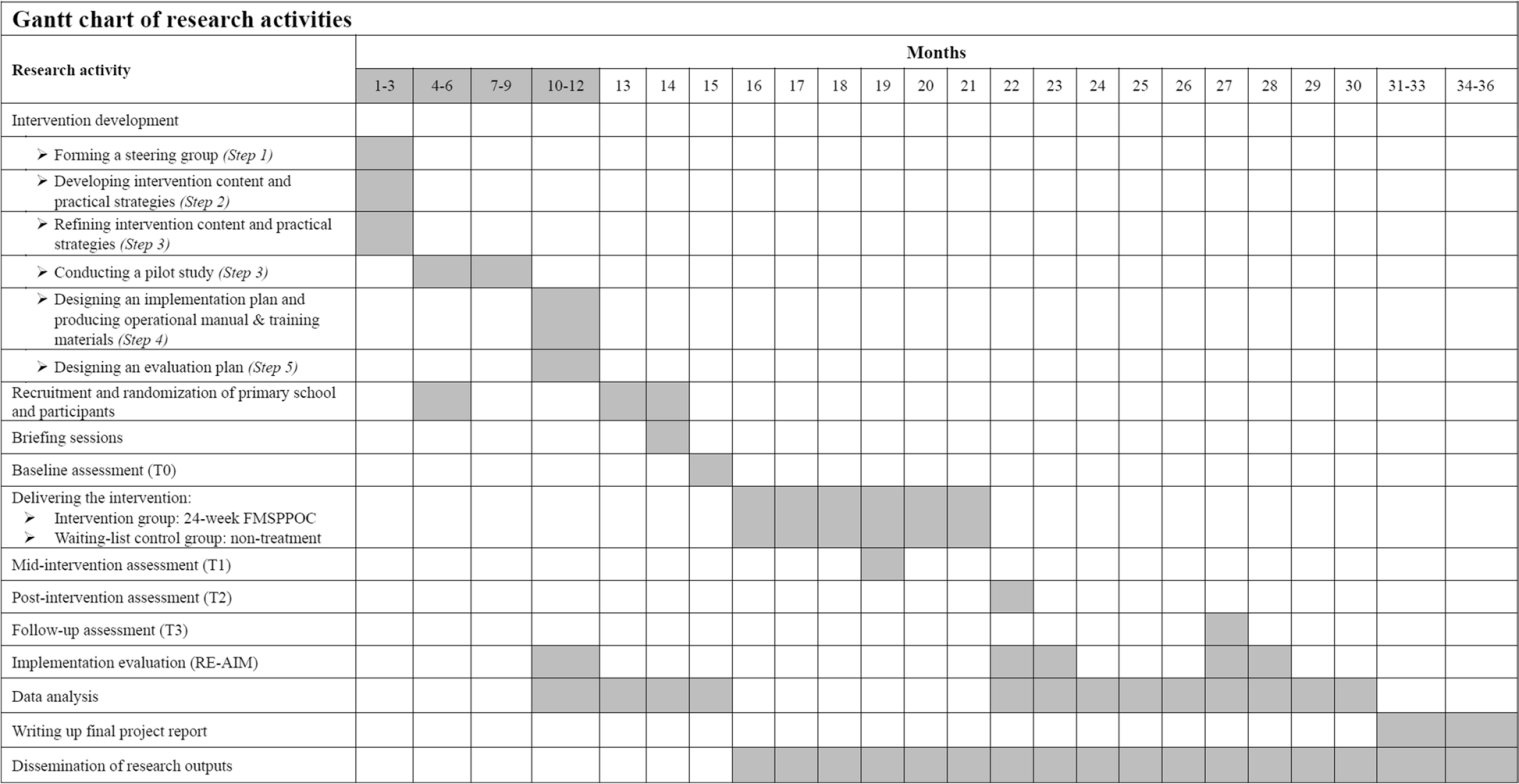
Gantt chart of research activities

The FMSPPOC development will be facilitated through five steps. Step 1 is to form a steering group, consisting of relevant stakeholders (e.g., research team members, PA instructors, primary school managers, and obese children and their parents). In Step 2, the research group will develop the intervention content and practical strategies. In the 3rd step of program production, all the stakeholders will discuss on consensus with respect to the refining and confirming of the intervention content and practical strategies, and then a two-month pilot study will be conducted to test and optimize the strategies and relevant materials. Participant recruitment and maintenance, data collection instrument and schedule, adaptability and feasibility of the intervention will be fully considered in the pilot study. In Step 4, a comprehensive plan for the intervention implementation will be constructed. To ensure the safety, supportiveness, enjoyment, and efficiency of the intervention during the implementation. An operational manual and training materials will be developed in this step. In Step 5, a plan to evaluate the implementation and effectiveness of the intervention will be developed. Any amendments will be made if necessary (Fig. 2).

For the project implementation, two 60-min face-to-face briefing sessions will be first provided for the intervention facilitators (i.e., PE teachers, student helpers, parents) one month prior to the FMSPPOC commencement (delivered by the investigators and/or research assistant) in the university gymnasium. One briefing session will focus on necessary knowledge and skills training so that PE teachers and student helpers can implement the intervention plan smoothly and effectively. In addition, the implementation details, rationale, nature, and benefits of FMSPPOC will be introduced. Another briefing session will train all evaluators on the measurement of FMSs, health behaviors, and physical fitness, as well as the specification of psychological measures.

For the main study, the school-based PA sessions will be conducted in an indoor playground at a primary school during afternoon custody time (4:00–5:00 p.m.) by qualified PE teachers with assistance of student helpers. The attendance of participants will be also recorded. In addition, to monitor the PA intensity, three mid-tests will be conducted using downloadable, wireless Polar Team Pro (Polar Team Pro, Kempele, Finland) and Polar heart rate sensor during the ball games (week 3, 7, & 11).

For the family-based PA assignments, relevant tasks and instructions will be introduced for parents in the beginning briefing session. The research assistants will remind parents to upload the required information each week via SMS messages and deliver the incentives correspondingly. For the second 12-week maintenance phase, the offline workshops will be implemented at a multi-function sports room in a primary school, while the online webinars will be conducted using Ding-Talk APP. These workshops and webinars will be facilitated by trained PE teachers and health experts from Hebei Normal University. Each participant will be asked to attend the workshops and webinars with at least one parent or legal guardian.

### Implementation evaluation

This study will use the Reach, Effectiveness, Adoption, Implementation, and Maintenance (RE-AIM) framework [74, 75] to assess the implementation of FMSPPOC among obese children. Details can be found in Table 3.

**Table 3:** The RE-AIM framework of implementation evaluation for the FMSPPOC program

Element	Program phase	Quantitative measures	Qualitative measures
**Reach**	Pilot study	1. The number of target participants who are willing to participate in the pilot study ● 20 obese children from a weight loss center for children and adolescents in Shijiazhuang, China	1. Identify the feasibility of recruiting enough eligible participants 2. Identify the barriers for recruitment and enrolment 3. Identify the facilitators and barriers for obese children 4. Identify the reasons for participants dropout 5. Identify the strategies for improving the adherence
	Main study	1. The number of target participants who are willing to participate in the main study ● Expected: 168 obese children from six primary schools of three districts (Yu-Hua, Chang-An and Xin-Hua; two schools per district) in Shijiazhuang, China	
**Effectiveness**	Main study	1. What proportion of participants completes the intervention? ● To ensure more than 85% participants completing the intervention program	1. Explore the experience of participating in the program from participants’ views 2. Identify the conditions and circumstances that influence the intervention effectiveness 3. Identify the reasons that result in the variations/differences in the intervention effects across participants within the program 4. Identify the potential contamination for the intervention effectiveness 5. Count numbers of sports injuries and other unintended consequences during the intervention period, and analyze the reasons
		2. How do completers compare to non-completers? ● Dropout analysis will be conducted to identify the baseline difference between completers and dropouts ● Sensitivity test will be conducted to identify the impact of the dropouts on the evaluation of the intervention effects	
		3. What are the effects of the intervention on participants? ● Significant improvement in primary outcome (FMSs) and other health-related physical and psychological outcomes, e.g., PA, fitness, wellbeing, perceived motor competence and PA enjoyment (detailed outcome measures are shown in Table 4) ● Significant changes in forming a regular PA habit ● Improved awareness and knowledge on the importance of PA habit and healthy lifestyles towards FMSs and obesity prevention	
**Adoption**	Pilot study & Main study	1. The proportion of service providers/collaborators (i.e., Shijiazhuang Education Bureau, Physical Fitness Association, Lab of Measurement and Evaluation in Health Sciences and Sports Nutrition Center, Hebei Normal University, and Department of Physical Education and Research and Department of Health Care in primary schools) involved in the program ● Expected: 4 service providers/collaborators will be involved in the pilot study ● Expected: 5 service providers/collaborators will be involved in the main study 2. How about the efficiency and quality of collaboration? ● Using self-reported scale to assess the satisfaction with efficiency and quality of collaboration among different service providers/collaborators	1. Identify facilitators and barriers that affect service providers/collaborators’ participation 2. Identify the facilitators and barriers that affect the efficiency and quality of collaboration
**Implementation**	Pilot study & Main study	1. Is the intervention delivered as intended? ● Using a process evaluation framework to assess the fidelity and quality of intervention implementation (e.g., exercise intensity will be monitored during the intervention; a fidelity evaluation scale will be used) ● Trained research staff observe teachers’ implementation of the FMSPPOC program (e.g., percent of lesson content conveyed, whether lessons will be presented in recommended order) and parents’ involvement in the family-based PA assignments ● Using self-reported e-logs to assess the participants’ satisfaction with the intervention implementation ● Using a self-reported scoring sheet to assess the consistency in delivering the intervention and evaluate the consistent commitment of facilitators and collaborators	1. Identify the acceptability, adaptability, and practicality of the intervention 2. Identify any modifications that have been done during the intervention process, and explore the reasons behind them 3. Identify the barriers that influence the fidelity of the intervention 4. Identify the potential facilitators and barriers that influence the intervention implementation and provide strategies to address them in the future
		2. Did participants adhere to the intervention program? ● Analyses for the attendance rate of each session and overall completion rate ● Check the completion of the family-based PA assignment by self-reported e-logs ● Self-reported e-logs completed by the intervention facilitators and collaborators	
**Maintenance**	Main study & Follow-up	1. The extent to which the intervention outcomes are sustained ● Data analyses for the sustained intervention effects during 6-month follow-up	1. Identify the components that influence the successful sustainability of the current program 2. Identify the modifications made to sustain the current program 3. Identify the facilitators and barriers of seeking more collaborators in future 4. Identify the facilitators and barriers that maintain the program and promote the program to a wider population in future
		2. The number of participants sustained, and the number of new participants enrolled in the program ● Expected: more than 85% of participants sustained and more than 300 new participants from five main districts (60 ~ 80 participants in each district) 3. The number of PE teachers and parents have sustained their implementation of the program or disseminated the program to others ● Expected: more than 70% of PE teachers and parents sustained the implementation of the program or introduce the program to others	
		3. The degree to which the Shijiazhuang Education Bureau institutionalize the FMSPPOC as an on-going part of their regular activities (i.e., supply their own funding, integrated into programmatic activities, regularly train their staff in implementation, continue to provide data for monitoring and evaluation) ● Expected: more than 70% of service providers/collaborators continue to implement the FMSPPOC program and for at least three years	
		4. The number of new service providers/collaborators, including but not limited to NGOs, sponsorships, and local government support, join the FMSPPOC ● Expected: more than 15 new collaborators from 5 main districts (3–5 per district)	

### Outcome evaluation

A description of the measurements in the present study are presented in Table 4. All the measurements will be conducted on four occasions, including baseline assessment (T0), mid-intervention assessment (T1: 12 weeks after the baseline assessment), post-intervention assessment (T2: 24 weeks after the baseline assessment), and follow-up assessment (T3: 6-month after the intervention completion).

**Table 4:** Description of the outcome measures

Outcomes	Measurement instrument
**Primary outcome: FMSs**	
Gross motor skills	Evaluated using The Test of Gross Motor Development-Third Edition (TGMD-3) which has been validated in China with satisfied reliability [76]. The TGMD-3 includes two sub-scales, the locomotor skill sub-scale composed of six skills: run, gallop, hop, horizontal jump, slide (judged on four performance criteria) and skip (judged on three criteria), and the ball skill sub-scale (previously named object control skill in the TGMD-2) composed of seven skills: one hand forehand strike of self-bounced tennis ball, kick a stationary ball, overhand throw, underhand throw, two hand strike of a stationary ball, one hand stationary dribble and two hand catch. Before the assessment of each skill, an accurate verbal description and demonstration of each skill was carried out by a trained researcher. Each child completed three trials, one for practice and then two formal trials. Only the scores of the two formal trials were recorded for the evaluation. Children’s performances were observed and evaluated following 3 ~ 5 qualitative performance criteria for each TGMD-3 assessment skill: every criterion was scored 1 point (present) or 0 point (absent) using process-oriented checklists [77]. The total score for each item is given by the sum of both trials. Items' sums were used to calculate the score for the locomotor (46) and ball control skills sub scales (54) as well as for the overall TGMD-3 scores (100) [77]
Manual dexterity and balance	Assessed using a subscale of the Movement Assessment Battery for Children-Second Edition (MABC-2, band 2 and 3) [78], which has demonstrated good reliability and validity in Chinese children [79]. Manual dexterity, which is composed of placing pegs, threading lace and drawing trail 2. Balance, which includes one-board balance, walking heel-to-toe forwards and hopping on mats. The scoring was consistent with the method published in the movement ABC-2 UK manual [78]
**Secondary outcomes**	
Health behaviors: PA, sedentary behavior, and sleep	The ActiGraph GT3X + accelerometer (ActiGraph LLC, Pensacola, FL, USA) will be used to objectively monitor whole-day PA. All participants will be asked to wear a monitor at the waist on an elasticized belt at the right midaxillary line. Participants were encouraged to wear the accelerometer 24 h per day (removing only for water-based activities: i.e., swimming/bathing) for at least 7 d, including two weekend days. Days with > 16 h/d of activity recordings (from midnight to midnight) were considered as valid [80], and the minimum amount of non-sleep data that was considered acceptable for inclusion was at least 4 days with at least 10 h of wake wear time per day, including at least one weekend day [81]. Data were collected at a sampling rate of 80 Hz downloaded in 1-s epochs with the low-frequency extension filter using the ActiLife software version 6.13 (ActiGraph LLC) and reintegrated to 15-s epochs for analysis. Non-wear time will be defined as a period of 20 consecutive minutes or more zero counts [82]. Night sleep duration was calculated in R software using GGIR package (version 2.0) default algorithm, as described by Van Hees et al. [83]. Evenson cut-off points [82] will be applied to determine non-sleep time spent in light (25–574 counts/15-s), moderate (574–1003 counts/15-s) and vigorous PA (> 1003 counts/15-s), and total sedentary time as all movement ≤ 25 counts per 15 s. Parents will be instructed to fill in sleep logs for their child with the purpose of cross-validating the waking (wear) time
Physical fitness	Assessed using the revised 2014 version of the Chinese National Student Physical Fitness Standard (CNSPFS) [84], involving a total of 11 physical fitness indicators. The 7 described below are suitable for primary school students, including BMI, vital capacity, 50 m sprint, Sit and reach, timed skipping rope, timed sit-ups (just Grades 3–6) and 50 m × 8 shuttle-run (just Grades 5 and 6). Following the guidelines, test examiners conducted each test per a protocol determined a priori. Each fitness indicator score was weighted by a grade- and sex-specific percentage
Perceived motor competence	Assessed using the subscale (athletic competence) of the Self-perception Profile for Children (SPPC) [85]. Athletic competence subscale of SPPC includes 6 items, three of the items are worded such that the first part of the statement reflects low competence or adequacy, and three are worded to first reflect high perceptions of competence or adequacy. This counterbalancing is reflected in the scoring of items, where half of the items are scored 1, 2, 3, 4 and half are scored 4, 3, 2, 1. This is to insure that children are tracking the content of the items and are not simply providing random response choices or are always checking the same side of all questions. In addition, the Chinese version of the SPPC has adequate reliability, ranging from 0.61 to 0.76 [86]. The structure and criterion validity are acceptable [87]
Perceived well-being	The Chinese version of the 12-item Psychological Well-Being Scale for Children (PWB-C) will be used [88, 89]. PWB-C contains six dimensions of psychological well-being: environmental mastery, personal growth, purpose in life, self-acceptance, autonomy, and positive relations with others. Options were given on a 4-point Likert scale, ranging from 1 (“almost never”) to 4 (“very frequently”). The mean score of the 12 items will be calculated, with a higher score indicating a higher level of perceived well-being
M-PAC components of PA for both parents and children	The Chinese translated items of M-PAC components of PA will be used [90–92]. The questionnaire package includes measures for behavioral intention, instrumental and affective attitudes, perceived capability, perceived opportunity, parental support (intentional and actual), action planning and coping planning, action control, habit strength, and identity. The response options and scoring approach will be consistent with the settings in previous studies [65, 66, 90, 91]
Anthropometric and body composition measures	Height and weight will be measured calibrated medical digital scales (RGT-140, Changzhou, China) and portable stadiometer (GMCS-I, Beijing, China) to the closest 0.05 kg and 0.1 cm, respectively following a standardized protocol [84]. Waist circumference will be measured using a flexible plastic tape at 1 cm above the umbilicus from the horizontal level in a standing position, at the end of a normal expiration [92]. Each of aforementioned anthropometric indexes was measured twice and the mean value was used for data analysis. A Bio-Impedance Analysis (BIA) was conducted using Portable body composition analyzer (InBody230, Seoul, South Korea) and Lokin Body 120 software (DMS-BIA technology; InBody Co., Seoul, South Korea) to estimate body composition, including percent of body fat (PBF), fat mass (FM), fat-free mass (FFM, kg) and skeletal muscle mass (SMM, kg). The instrument was validated against dual-energy X-ray absorptiometry for school-age children with satisfactory results for estimating body fat [93]
**Additional information**	
Demographics	Children’s age, gender, grade (primaries 1–6), ethnicity (Han or others), parental educational level (below college; college or above) and yearly household income (low: RMB < 84,000; medium: RMB 84,000 – 132,000; high: RMB > 132,000) [60] will be reported by parents

In addition, the collection of all indicators at the four-time points will follow the following process. Day 1–7: Demographic information (filled by parents) and ActiGraph 3X + measured PA, sedentary behavior, and sleep; Day 8: anthropometric and body composition measures and FMSs; Day 9: physical fitness assessment; and Day 10: psychological outcomes. The Day 8–10 data collection will be implemented by four trained student helpers with assistance of PE teachers in an elementary school gymnasium, where the temperature will be constant at 20℃ and humidity will be controlled at 50% during the assessment.

### Data analysis

Quantitative data analyses will be performed using SPSS and Mplus. Descriptive statistics of all continuous variables will be expressed as mean ± standard deviation (*M* ± *SD*). Independent samples *t*-tests and Chi-square tests will be employed for checking the randomization, analyzing the dropout, and detecting the potential confounders at baseline. Intention-to-treat approach will be used for the primary analyses, with per-protocol analyses as sensitivity tests. Missing data will be imputed using a multiple imputation approach with linear and logistic regression equations. The statistical significance level will be set to *p* < 0.05 (two-tailed). Generalized linear mixed models (GLMM) will be used to evaluate the intervention effects on outcome measures, with time, condition, and the interaction of time and condition as fixed effects, adjusting for baseline values. Post-hoc tests will be conducted where a significant interaction effect is detected by using the least significant difference method. In addition, structural equation modeling will be used to analyze the interrelationship (e.g., mediation mechanisms) of outcomes, using a bias-corrected bootstrap approach (5000 resamples). For the qualitative data, a thematic analysis approach will be applied using NVivo 11 software.

## Discussion

Obesity is becoming an increasing problem also in China and Chinese children, but rather little evidence is available how to address this. There are indicators, that this trend is similar to those in other countries. In 2008, Stodden et al. [33] proposed a “conceptual model of children's motor development” based on partial evidence and experience, which hypothesized the relationship between FMSs and multiple health indicators (e.g., PA, perceived motor competence, physical fitness, and weight status) of children. The authors point out that FMSs levels may positively or negatively affect PA and weight status in children, and that healthy or unhealthy weight status may also promote or restrict the development of FMSs in children over time, with perceived motor competence and physical fitness mediating the relationship between FMSs and PA [33]. In recent years, overweight and obese children have been consistently reported as scoring significantly lower than their healthy weight counterparts on FMSs, suggesting that poor FMSs can contribute to overweight/obesity [21–25, 94]. Similarly, obesity may likewise act as a constraint on FMS development and proficiency, generating biomechanical changes and adjustments in movement [94]. In previous studies, FMSs have been shown to correlate with PA in school-aged children [4], and strong positive associations have been observed between FMS proficiency in children at age 6 and leisure time PA in adults at age 26 [95]. Further to regular PA participation, additional health benefits of FMSs proficiency have been associated with increased cardiorespiratory fitness [96] and perceived motor competence [10, 14], as well as reduced overweight and obesity [12, 13]. This indicates the importance of effective interventions being implemented to allow overweight/obese children to develop their FMS early, reducing their risk of obesity through continued PA into adolescence and adulthood.

To our knowledge, FMSPPOC is the first school-family blended multi-component FMS-promotion program to be designed in China, which makes up the research and practical gaps as suggested in previous studies and shows a series of strengths: 1) standard methods will be applied for assessment of FMSs; 2) the use of theoretical framework and behavioral change techniques will ensure the implementation and effectiveness of the FMSs promotion program; 3) the gold standard for scientific designs (i.e., CRCT) will be applied and comprehensive measures will be implemented, which can contribute to a robust examination of the program effectiveness and a better understanding of the potential mechanisms; 4) the use of the RE-AIM framework will enhance the quality of the program and enable us to evaluate the implications of FMSPPOC among obese children broadly. We anticipate that FMSPPOC will also be a new paradigm of secondary obesity prevention. In addition, we will propose and assist health and education governments to advocate and disseminate the blended intervention among all primary schools to tackle unhealthy lifestyles in children and further improve health status of obese children in China. Other countries and regions with similar demographics to China can also learn and benefit from this product. Surely, comparisons to other cultures need to follow up and mechanisms are required to be evaluated, too. With that, the research of this study and comparable one can inform not only research and practice but also theory refinement and scaling up approaches.

## Supplementary Information

**Additional file 1.**

## Data Availability

Requests of future data and developed materials should be directed to the corresponding author or first authors.
